# Silicon Regulates Antioxidant Activities of Crop Plants under Abiotic-Induced Oxidative Stress: A Review

**DOI:** 10.3389/fpls.2017.00510

**Published:** 2017-04-06

**Authors:** Yoon-Ha Kim, Abdul L. Khan, Muhammad Waqas, In-Jung Lee

**Affiliations:** ^1^Division of Plant Biosciences, Kyungpook National UniversityDaegu, South Korea; ^2^Division of Plant Sciences, University of Missouri-ColumbiaColumbia, MO, USA; ^3^UoN Chair of Oman's Medicinal Plants and Marine Natural Products, University of NizwaNizwa, Oman; ^4^Department of Agriculture, Abdul Wali Khan University MardanKhyber Pakhtunkhwa, Pakistan

**Keywords:** oxidative stress, stress response, Si fertilization, biochemical and physiological function, stress in plants

## Abstract

Silicon (Si) is the second most abundant element in soil, where its availability to plants can exhilarate to 10% of total dry weight of the plant. Si accumulation/transport occurs in the upward direction, and has been identified in several crop plants. Si application has been known to ameliorate plant growth and development during normal and stressful conditions over past two-decades. During abiotic (salinity, drought, thermal, and heavy metal etc) stress, one of the immediate responses by plant is the generation of reactive oxygen species (ROS), such as singlet oxygen (^1^O_2_), superoxide (${\text{O}}_{2}^{-}$), hydrogen peroxide (H_2_O_2_), and hydroxyl radicals (OH), which cause severe damage to the cell structure, organelles, and functions. To alleviate and repair this damage, plants have developed a complex antioxidant system to maintain homeostasis through non-enzymatic (carotenoids, tocopherols, ascorbate, and glutathione) and enzymatic antioxidants [superoxide dismutase (SOD), catalase (CAT), and ascorbate peroxidase (APX)]. To this end, the exogenous application of Si has been found to induce stress tolerance by regulating the generation of ROS, reducing electrolytic leakage, and malondialdehyde (MDA) contents, and immobilizing and reducing the uptake of toxic ions like Na, under stressful conditions. However, the interaction of Si and plant antioxidant enzyme system remains poorly understood, and further in-depth analyses at the transcriptomic level are needed to understand the mechanisms responsible for the Si-mediated regulation of stress responses.

## Introduction

Silicon (Si) has a strong affinity with oxygen; therefore, it usually exists as silica (SiO_2_) under natural conditions (Ma and Takahashi, 2002). It also exists in the form of silicic acid [Si(OH)_4_] and silicate (x${\text{M}}_{2}^{1}$OySiO_2_), depending upon the soil pH (Epstein, 1999). Si accumulation/transport occurs in the upward direction, and has been identified in several crop plants. Si transporter genes have been identified in rice, barley, and maize roots, which facilitate its absorption from the soil to the shoot area. Subsequently, it stimulates various physiological responses such as growth, development, and optimization of enzymatic activities. Si accumulation in plant normally occurs from the root to shoot, and its transport process has been identified in several crops such as rice, maize, and barley (Ma et al., 2006; Mitani et al., 2009; Cooke and Leishman, 2011; Yamaji et al., 2012). Two Si transporter genes were identified by Ma et al. (2006) in rice root and was named as low silicon gene 1 (*Lsi1*) and low silicon gene 2 (*Lsi2*). Following this several Si transport genes have been characterized in other corps such as barley (*HvLsi1, HvLsi2*) and maize (*ZmLsi1, ZmLsi2*; Ma et al., 2006; Mitani et al., 2009; Yamaji et al., 2012). After the absorption of Si from the soil into the root, it gets translocated to the shoot area, where it can stimulate various physiological responses, such as plant growth and development (Epstein, 1999; Hamayun et al., 2010; Kim et al., 2012; Mateos-Naranjo et al., 2015), enzymatic activity (Epstein, 1999; Liang et al., 2003; Gong et al., 2005; Kim et al., 2014a,b,c; Todorova et al., 2014; Abdel-Haliem et al., 2017), and gene expression (Ma and Yamaji, 2008; Kim et al., 2014a; Vatansever et al., 2017).

To complete a life cycle, plants are continuously exposed to various abiotic stresses and sometime multifaceted stresses. These stresses in turn causing the generation of various reactive oxygen species (ROS), such as singlet oxygen (^1^O_2_), superoxide (${\text{O}}_{2}^{-}$), hydrogen peroxide (H_2_O_2_), or hydroxyl radicals (OH) in cells (Sharma et al., 2012; Das and Roychoudhury, 2014). These ROS can cause serious oxidative damage to the protein, DNA, and lipids of cell components (Apel and Hirt, 2004; Lobo et al., 2010; Tripathi et al., 2017). Therefore, ROS scavenging is most important defense mechanism to cope with stress condition in plants (Sharma et al., 2012; Baxter et al., 2014; Das and Roychoudhury, 2014). According to previous reports, exogenously Si can improve the ability of ROS scavenging by regulation of antioxidants enzyme activity (Torabi et al., 2015; Kim et al., 2016; Tripathi et al., 2017). Furthermore, regulation pattern across various crop plants is different depending upon the exposure time of the stress (Sharma et al., 2012; Kim et al., 2016). Therefore, here, we discussed various possibilities based on previous literature survey and our understanding the role of Si in modulating antioxidant activities in plants during abiotic stress.

## Defense mechanism against ROS generation

In natural conditions, plants continuously produce several ROS during photosynthesis and respiration processes in cell organelles such as mitochondria, chloroplast, and peroxisomes. Thus, plants can maintain homeostasis by two different detoxification mechanisms involving non-enzymatic and enzymatic antioxidants (Mittler, 2002; Arbona et al., 2003; Apel and Hirt, 2004; Sytar et al., 2013; Wu et al., 2017). In plants, superoxide dismutase (SOD), catalase (CAT), and ascorbate peroxidase (APX) are the main enzymatic antioxidants, whereas carotenoids, tocopherols, ascorbate, and glutathione are classified as the non-enzymatic antioxidants (Asada, 1999; Racchi, 2013; Kim et al., 2014b,c). Racchi (2013) reported that SOD exists in various forms, such as Cu/ZnSOD, MnSOD, and FeSOD. Depending upon their affinity with the other ions in plants, each SOD are distributed in a different form in various plant organs such as chloroplasts (Cu/ZnSOD, FeSOD), cytosol (Cu/ZnSOD), and mitochondria (MnSOD). Primarily, SOD catalyzes the efficient removal of superoxide free radicals in chloroplasts as they are mainly generated in the photosystem I during the light reaction. CAT is located in the peroxisomes of plant cells, and its main role is the elimination of H_2_O_2_, which is produced by the SOD reaction. Another antioxidant, APX, also can remove H_2_O_2_; however, it is distributed in the preoxisomes as well as chloroplasts, cytosol, and mitochondrion (Racchi, 2013). Thus, APX can be found in different forms, such as cAPX (cytosol), mitAPX (mitochondria), sAPX (chloroplast stroma), mAPX (peroxisomes and glyoxisomes), and tAPX (chloroplast thylakoids) depending upon its location (Racchi, 2013). In the chloroplast, APX exist as sAPX and tAPX; the ratio of sAPX and tAPX in chloroplast differs according to the plant species and leaf senescence, and reveals different plant sizes (Sun et al., 2010). The cAPX is located in cytosol; thus, its plays a role in the elimination of H_2_O_2_, which is generated in cytosol. Therefore, all APXs are different in characteristics such as size, location, role, and amino acid sequences (Caverzan et al., 2012).

**Figure 1:**
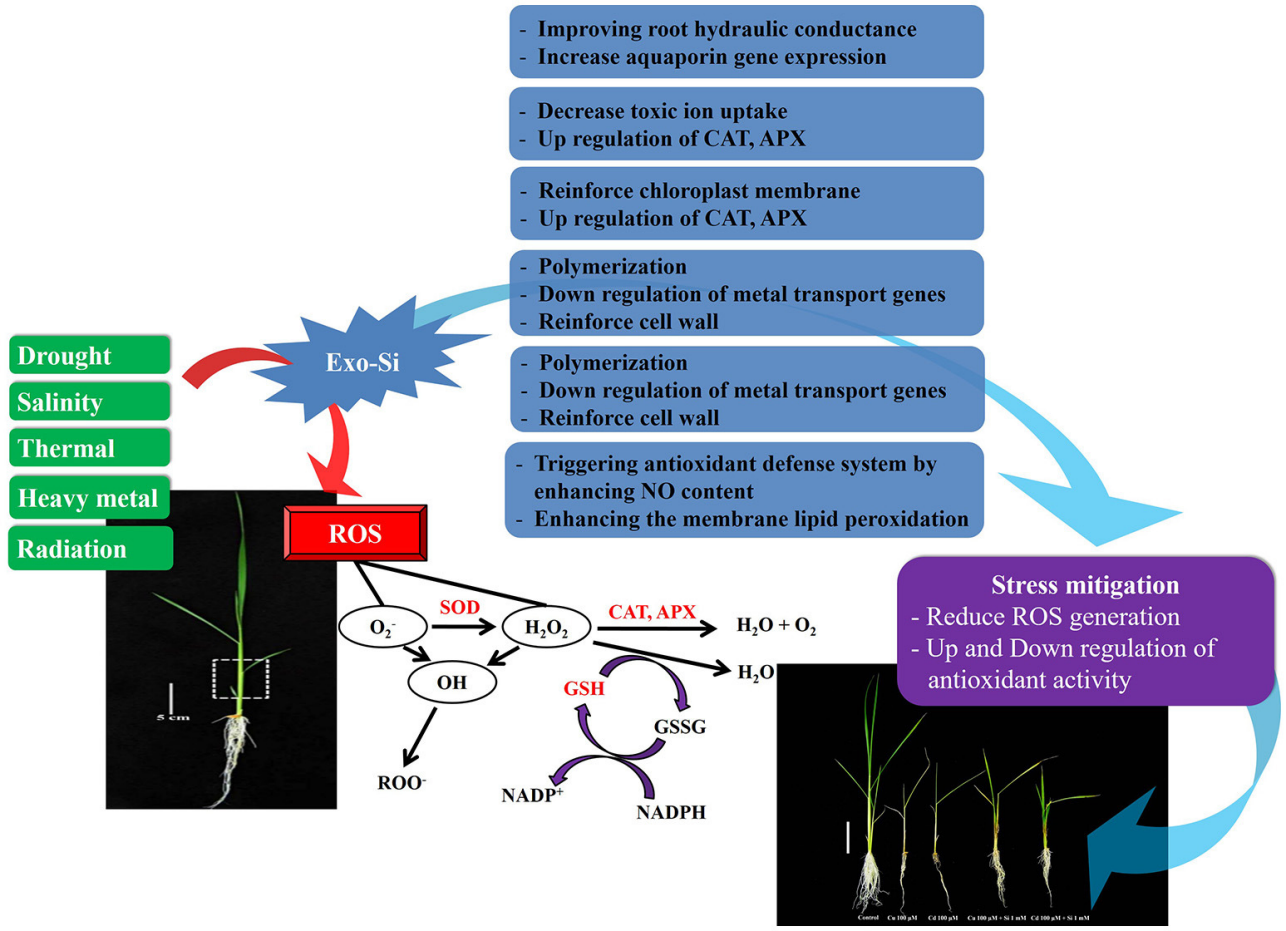
**Schematic presentation shows the possible causes that overproduce the reactive oxygen species that could disturb the normal function of cells**. The mechanism of antioxidants shown here scavenges the ROS as well as Si effect to mitigate abiotic stress condition.

Plants can induce defense responses against oxidative stress by activating the non-enzymatic antioxidants, which represent the second line of defense against ROS, hydrophilic molecules (ascorbate, glutathione), and lipophilic metabolites (carotenoids, α-tocopherol; Racchi, 2013; Suzuki et al., 2014; Gowayed et al., 2017) Ascorbate is a water-soluble antioxidant synthesized in mitochondria. It can translocate to other cell compartments by two different pathways. Normally, ascorbate can directly scavenge ROS (^1^O_2_, ${\text{O}}_{2}^{-}$, and OH) in the cell. Furthermore, it is connected with the de-epoxidase enzyme of violaxanthin, and acts as response matrix of APX (Szarka et al., 2013). Due to its various roles, ascorbate is considered as the most powerful antioxidant in the plant cell (Gill and Tuteja, 2010; Racchi, 2013; Suzuki et al., 2014). Glutathione is also an important water-soluble antioxidant, and plays an important role in scavenging ^1^O_2_ and OH from chloroplasts (Sharma et al., 2012). In addition, glutathione protects the thiol-groups of enzymes located in the chloroplast stroma and participates in the production of α-tocopherol and ascorbate (Xiang and Oliver, 1998; Hicks et al., 2007; Sharma et al., 2012; Racchi, 2013). Besides its role in detoxification of ROS, glutathione induces physiological responses such as the regulation of sulfur transport and expression of stress defense genes (Noctor et al., 2002; Racchi, 2013). Carotenoids are a class of phenolic compounds distributed in various fruits and vegetables (Racchi, 2013). They can prevent lipid peroxidation by scavenging single oxide radical from chloroplasts (Kühlbrandt et al., 1994). Carotenoids are synthesized in plastids and consist of 40-carbon isoprenoids. According to Lu and Li (2008), carotenoids are classified as carotenes, which include the carbon and hydrogen atoms, and xanthophylls that contain the oxygenated form of carotenes (Wu et al., 2017). The most important role of α-tocopherol is that it can eliminate ^1^O_2_, ${\text{O}}_{2}^{-}$, and OH free radicals, which are generated in the thylakoid membranes; thus, it can prevent lipid peroxidation (Fryer, 1992; Kataria, 2017). α-tocopherol has adequate fluidity, enabling it to move easily within the lipid membrane. Thus, membrane safety is induced by the fluidity of α-tocopherol (Faltin et al., 2010; Racchi, 2013; Figure 1).

## Si in ROS scavenging under abiotic stress conditions

The enzymatic/non-enzymatic antioxidants are involved in the removal of ROS either directly (catalases and peroxidases) or indirectly through the regeneration of the two major redox molecules (ascorbate and glutathione) in the cells (Figure 1). Accumulation of these antioxidants suggests a high level of stress convened to the plants (Sharma et al., 2012). This could be also assumed that the plant has defended itself from ROS by producing high amount of antioxidant/enzymes. Accoring to Rouhier and Jacquot (2008), Si application in crops during abiotic stress conditions can regulate ROS generation. Here, we investigated via past and current reports that how antioxidant enzymes could regulate after exogenously Si to different plant species during some of the common abiotic stresses (salinity, drought, temperature, wounding, UV, and heavy metal stress).

## Salinity stress

Decrease in water potential due to high concentration of sodium and chloride ions inhibit plant growth and development (Torabi et al., 2015). According to Kim et al. (2014c), the application of Si in rice plants under salinity significantly decreased the activities of non-enzymatic MDA and enzymatic antioxidants POD, PPO, and CAT on the other hands, Torabi et al. (2015) observed that when they applied Si to borage plant, SOD activity was significantly increased in Si treatment but activity of CAT and APX was slightly decreased in Si application (Table 1). However, Shekari et al. (in press) found that activities of CAT, APX, SOD, and POD were highly increased under Si application with NaCl to herbal *Anethum graveolens* plants. Same pattern of SOD, GPX, APX, GR, and CAT activities was observed by Al-aghabary et al. (2005); Liang et al. (2003), and Zhu et al. (2004). The activities were significantly increased in Si applied barley, cucumber, and tomato plants (Table 1).

**Table 1:** **Modulation of antioxidant activities by Si application under various abiotic stresses**.

**Abiotic stresses**	**Silicon effect**	**Crop plant**	**References**
Salinity	Increased activity of LPO	Barley	Liang et al., 2003
Salinity	Increased activity of SOD, GPX, APX, and GR Decreased activity of ELP and LPO	Cucumber	Zhu et al., 2004
Salinity	Increased activity of SOD and CAT Decreased activity of APX and MDA	Tomato	Al-aghabary et al., 2005
Salinity	Decreased activity of CAT, MDA, POD, and PPO	Rice	Kim et al., 2014c
Salinity	Increased activity of SOD Decreased activity of CAT and APX	Borago	Torabi et al., 2015
Salinity	Increased activity of CAT, APX, SOD, and POD	Dill	Shekari et al., in press
Drought	Increased activity of CAT, SOD, and GR	Wheat	Gong et al., 2005
Drought	Increased ascorbate contents Reduced glutathione and flavonoid contents	Wheat	Ma et al., 2016
Drought	Decreased activity of APX and MDA	Sunflower	Gunes et al., 2008
Drought	Increased activity of SOD and CAT Decreased activity of POD	Tomato	Shi et al., 2014
Drought	Increased activity of SOD and CAT	Tomato	Shi et al., 2016
High Tem.	Increased activity of SOD, APX, and GPX Decreased activity of CAT	*Salvia splendens*	Soundararajan et al., 2014
Low Tem.	Increased activity of SOD, GSH, APX, MDHAR, GR, and AsA Decreased activity of MDA	Cucumber	Liu et al., 2009
Low Tem.	Increased activity of GSH and AsA Decreased activity of MDA	Maize	Habibi, 2016
Low Tem.	Increased activity of SOD, CAT, and POD Decreased activity of MDA	Turfgrass	He et al., 2010
Mechanical Wounding	Increased activity of CAT, POD and PPO Decreased activity of MDA	Rice	Kim et al., 2016
Ultraviolet-B	Decreased activity of CAT and POD	Soybean	Shen et al., 2010
Ultraviolet-B	Increased activity of SOD and APX Decreased activity of CAT and GPX	Wheat	Tripathi et al., 2017
Heavy metal (Cd)	Decreased activity of MDA	Rice	Kim et al., 2014a
Heavy metal (Mn)	Decreased activity of POD	Cucumber	Maksimović et al., 2012
Heavy metal (Cr)	Increased activity of SOD, GR, and CAT Decreased activity of APX	Pea	Tripathi et al., 2015

*APX, ascorbate peroxidase; CAT, catalase; GSH, glutathione reduced form; GR, glutathione reductase; GPX, guaiacol peroxidase; LPO, lipid peroxidase; SOD, superoxide dismutase; ELP, electrolytic leakage percentage; MDA, malondialdehyde; POD, peroxidase; PPO, polyphenol peroxidase; MDHAR, monodehydroascorbate reductase; AsA, ascorbate*.

## Drought stress

Drought condition cause damage the photosynthetic pigments and disturb balance between ROS production and antioxidants thus overall affecting crop productivity (Iturbe-Ormaetxe et al., 1998; Gong et al., 2005). According to Gong et al. (2005), Si treatment in wheat plants caused high drought tolerance by up-regulating antioxidant activities of CAT, SOD, and GR (Table 1). Ma et al. (2016) also suggested that Si supplement wheat plant showed lower lipid peroxidation, glutathione and total flavonoid content whereas increased ascorbate content was observed. Similarly, Shi et al. (2014, 2016) reported that Si supplementation in tomato plants under PEG induced drought stress caused tolerance via increased SOD and CAT activities as well as improved water uptake ability of roots (Table 1). Whilst, Gunes et al. (2008) observed that Si decreased MDA and APX activities in sunflower during drought condition (Table 1).

## Thermal stress

Like other abiotic stress factors, thermo (cold and heat) stress may also disturb the balance between ROS and antioxidants activity. Soundararajan et al. (2014) treated *Salvia splendens* with Si under high temperature (35°C), and found that the activities of SOD, APX and GPX were increased and contrarily that of CAT was decreased (Table 1). Liu et al. (2009) observed that during low temperature (day/night; 15/8°C), Si applied to hydroponically cultivated cucumber plant were more resistant to chilling stress compared to non-Si application and was attributed to more activated antioxidants such as SOD, GSH, APX, GR, MDHAR, and AsA (Table 1). Almost same tendency of chilling (day/night; 15/5°C) stress tolerance was observed in turf grass as well, after Na_2_SiO_3_ fertilization into soil (He et al., 2010; Habibi, 2016; Table 1).

## Mechanical wounding

Normally, natural wounding stress is caused by herbivory or lodging and these events could increase hydrogen peroxide level inside plant tissues (León et al., 2001). According to Kim et al. (2014a), exogenous Si application in rice plants improved mechanical strength to overcome losses from wounding stress (Table 1).

## Ultraviolet-B

Many studies demonstrated that Si application can induce resistance to UV-B stress via physiological and biochemical process in plants (Tripathi et al., 2017). In particular, when UV-B applied to tropical plants, MDA, POD, SOD, and anthocyanin contents was increased however, they found decreased activity of CAT was measured (Todorova et al., 2014; Table 1). According to Tripathi et al. (2017), UV stress was significantly improved at Si and Si nanoparticle (SiNp) applied wheat seedlings. Especially, mitigation effects between Si and SiNp showed that SiNp applied wheat seedling revealed more strong resistance to UV-B stress (Table 1). Other study reported that decreased activity of POD and CAT were measured when they applied Si with UV-B stress to soybean plants (Shen et al., 2010; Table 1).

## Heavy metal stress

In addition, during heavy metal stress, Si application can regulate metal transport and prevent damage shown by decreased MDA activity in rice plants (Kim et al., 2016). In cucumber, Si application can ameliorate manganese toxicity observed by decreased POD activity (Maksimović et al., 2012; Table 1). Tripathi et al. (2015) applied SiNp with chromium (Cr) to pea seedling after that, they confirmed stress tolerance phenotypes such as enhanced photosynthetic pigments as well as increased activity of SOD, GR, and CAT however, APX activity was decreased (Table 1).

## Conclusions

During abiotic stress conditions, the Si application shows varying response to ROS scavenging by activating the defense system plants. In doing so, the activity of antioxidant arsenals (CAT, SOD, PPO, POD, APX, GPX, and GSSH) may also oscillate depending upon the intensity of stress and plant type. Si supplemented plants showed resistance to abiotic stress through, lowering ROS production by (i) enhancing CAT and APX activities as both are involved in conversion of H_2_O_2_ into H_2_O (ii) and decreasing MDA activity.

## Author contributions

YK wrote the manuscript; MW and AK contributed in drafting and revising manuscript; IL draw the figure and revised the manuscript.

## Funding

This work was supported by Korea Institute of Planning and Evaluation for Technology in Food, Agriculture, Forestry and Fisheries (IPET) through Agriculture, Food and Rural Affairs Research Center Support Program, funded by Ministry of Agriculture, Food and Rural Affairs (MAFRA) (716001-7)

### Conflict of interest statement

The authors declare that the research was conducted in the absence of any commercial or financial relationships that could be construed as a potential conflict of interest.
